# Tumor Hemodynamics and Hepatocarcinogenesis: Radio-Pathological Correlations and Outcomes of Carcinogenic Hepatocyte Nodules

**DOI:** 10.1155/2014/607628

**Published:** 2014-03-03

**Authors:** Kazuhiko Ueda, Osamu Matsui, Azusa Kitao, Satoshi Kobayashi, Jun Nakayama, Shinich Miyagawa, Masumi Kadoya

**Affiliations:** ^1^Department of Radiology, Shinshu University School of Medicine, 3-1-1 Asahi, Matsumoto 390-8621, Japan; ^2^Department of Radiology, School of Medicine Kanazawa University, 13-1 Takara-machi, Kanazawa 920-8641, Japan; ^3^Department of Molecular Pathology, Shinshu University School of Medicine, 3-1-1 Asahi, Matsumoto 390-8621, Japan; ^4^Department of Surgery, Shinshu University School of Medicine, 3-1-1 Asahi, Matsumoto 390-8621, Japan

## Abstract

Tumor hemodynamics of carcinogenic hepatocytes nodules, that is, low grade dysplastic nodules, high grade dysplastic nodules, early hepatocellular carcinomas (HCCs), and progressed HCCs, change during multistep dedifferentiation of the nodules. Morphometric analyses of inflow vessels of these nodules indicate that the portal veins of carcinogenic hepatocyte nodules monotonically decrease whereas the arteries bitonically change, first decrease and then increase. Findings on imaging techniques depicting these changes in tumor blood inflows, especially intra-arterial contrast-enhanced computed tomography, closely related not only to the histological differentiation of the nodules but also to the outcomes of the nodules. Histological analyses of connections between the vessels within the tumors and those in the surrounding livers and findings on imaging techniques indicate that drainage vessels of HCC change from hepatic veins to hepatic sinusoids and then to portal veins during multistep hepatocarcinogenesis. Understanding of tumor hemodynamics through radio-pathological correlations will be helpful in drawing up therapeutic strategies for carcinogenic hepatocyte nodules arising in cirrhosis.

## 1. Introduction

A stepwise model of development, from well-differentiated precursors to poorly differentiated progressed hepatocellular carcinomas (HCCs), is well established by evidences accumulated in the past three decades. In 1995 and 2009, international reproducible criteria for the diagnosis of nodular lesions of hepatocarcinogenesis were developed by the remarkable endeavors of the East and the West pathologists. At present, the criteria involve not only pathologic but also radiologic features, especially hemodynamic findings. For example, progressed HCCs are defined as radiologically hypervascular lesions without portovenous supply which histologically appear moderately or poorly differentiated; early HCCs and dysplastic nodules are defined as iso- or hypovascular lesions with portovenous supply on radiological images which appear well differentiated on histology [1, 2]. Thus, an intimate knowledge of the relations between tumor hemodynamics and hepatocarcinogenesis would be useful for the management of carcinogenic nodules as well as for the better understanding of multistep models of hepatocarcinogenesis. This review focuses on radiologic hemodynamic features of carcinogenic hepatocyte nodules arising from cirrhotic livers, that is, dysplastic nodules, early HCCs, and progressed HCCs, paying close attention to radio-pathological correlations and outcomes of the nodules.

## 2. Tumor Blood Inflows and Hepatocarcinogenesis

### 2.1. Pathology of Tumor Blood Inflows

**Figure 1:**
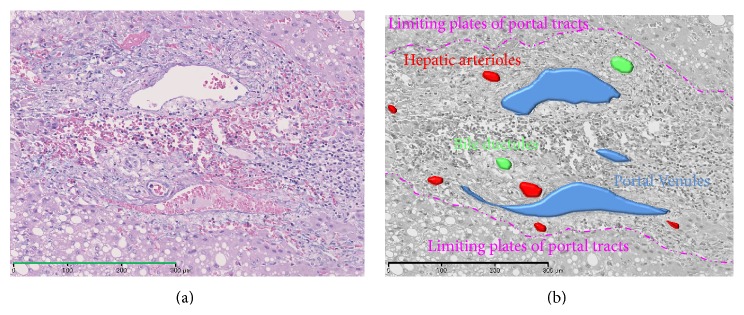
Photomicrograph of hepatic arteries and portal venules as inflow vessels in a portal tract within an early hepatocellular carcinoma stained with hematoxylin, eosin,and Victoria blue (Bar = 300 *μ*m) (a) and converted monochrome image of the same photomicrograph in conjunction with annotations of colored vessels, bile ductules, and limiting plates of the portal tract (b).

**Figure 2:**
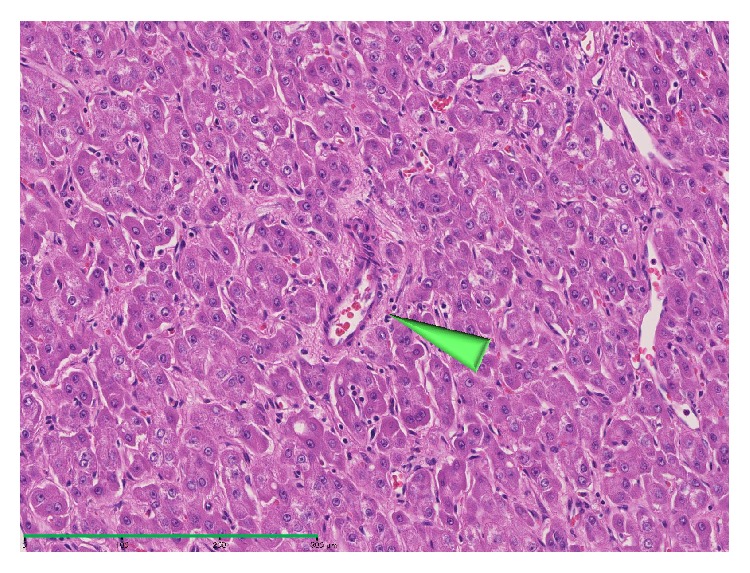
Photomicrograph of an unpaired artery unaccompanied by any bile ducts within a progressed hepatocellular carcinoma (arrowhead, hematoxylin and eosin, Bar = 300 *μ*m).

On histology, the blood circulation is not kinetically depicted, but tumor blood inflows can still be analyzed through the quantification of the inflow vessels with morphometric technique. The pathology of inflows of carcinogenic hepatocyte nodules has been morphometrically evaluated by two methods:counting the number of inflows vessels and measuring the luminal area of them. There are three types of inflow vessels of carcinogenic hepatocyte nodules—portal vein, hepatic artery, and abnormal artery which are termed unpaired or nontriadal arteries (Figures 1 and 2) [3–10]. The former two ones, portal vein and hepatic artery, are accompanied by bile ducts running within the portal tracts, whereas the last, unpaired artery, is not accompanied by bile ducts or portal veins independently running outside of portal tracts. It is now considered that the unpaired arteries are new vessels developed through neovascularization during hepatocarcinogenesis [3–10].

The inflow vessels in the carcinogenic nodules of the liver have been histomorphometrically quantified by counting the number of vessels in the unit area:vessel density. During dedifferentiation of the nodules, portovenous densities of the nodules decrease whereas arterial densities of the nodules increase [3–10]. The portovenous densities within dysplastic nodules are lower than those within the liver [3, 9].The arterial densities of low grade dysplastic nodules are almost as high as those of the surrounding livers; those of high grade dysplastic nodules are also almost as high as those of the livers; those of progressed HCCs are much higher than those of the livers. The proportions of the numbers of unpaired arteries to those of total arteries increase during the dedifferentiation.

**Figure 3:**
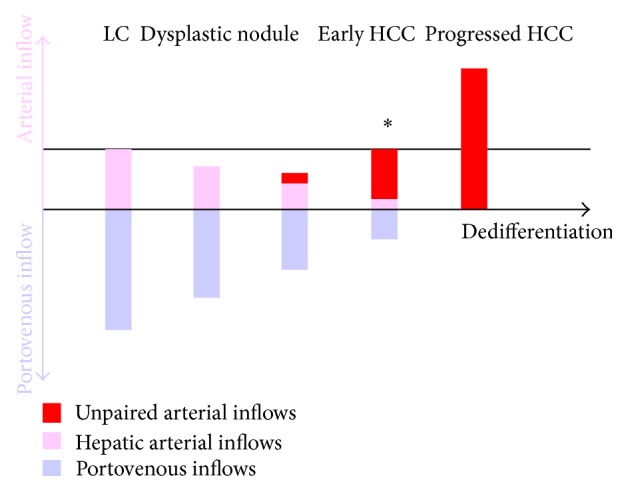
Outlines of measuring luminal areas of inflow vessels of carcinogenic hepatocyte nodules. Nodules with smaller portovenous areas and the same arterial areas to the surrounding livers (∗) are just on the final stage of high grade dysplastic nodules transiting to progressed HCCs.

Measuring luminal areas of the vessels is more recommendable than counting their number although the former is much more laborious than the latter, because luminal areas reflect angiography more accurately than numbers [3]. The portovenous luminal areas of low grade dysplastic nodules per unit area are as large as those of the surrounding livers; those of high grade dysplastic nodules are smaller than those of the livers; those of progressed HCCs are almost null. The arterial luminal areas of low grade dysplastic nodules per unit area are slightly smaller than those of the livers; those of high grade dysplastic nodules are smaller than those of the livers; those of progressed HCCs are much larger than those of the livers. The proportions of unpaired arteries to total arteries of low grade dysplastic nodules in luminal area are small, those of high grade dysplastic nodules are moderate, and those of progressed HCCs are almost 100%. To summarize, during the dedifferentiation, the portovenous areas of carcinogenic hepatocyte nodules monotonically decrease whereas the arterial areas bitonically change, first decrease because hepatic arterial areas decrease and then increase because unpaired arterial areas increase (Figure 3) [3]. Hepatocyte nodules with smaller portovenous areas and the same arterial areas to the surrounding livers are just on the final stage of high grade dysplastic nodules transiting to progressed HCCs [3].

### 2.2. Imaging Techniques for Evaluations of Tumor Blood Inflows

Tumor blood inflows, both portovenous and arterial, can be evaluated with imaging techniques. There are two techniques for evaluation of tumor portovenous inflows: Doppler sonography [11] and CT during arterial portography (CTAP) [12]. With Doppler sonography, inflows in some large portovenous branches in tumors may be visualized, but portovenous perfusions cannot be evaluated. With CTAP, tumor portovenous perfusions can be evaluated not only qualitatively but semiquantitatively [13–15]. For example, we can tell that a portovenous perfusion of a nodule is null or reduced. In addition, we can identify the perfusion difference between nodules and the surrounding livers. Moreover, with CTAP, portovenous perfusions can be determined separately from arterial perfusions. Thus, CTAP should be applied in patients with carcinogenic hepatocyte nodules as principal technique to confirm the portovenous inflows of the nodules.

There are six imaging techniques for evaluation of tumor arterial inflows: Doppler sonography as noncontrast imaging [11, 16], contrast sonography with perfluorobutane microbubbles [17–19], dynamic contrast-enhanced CT and dynamic contrast-enhanced MR imaging as intravenous contrast enhanced imaging, sonography with carbon dioxide microbubbles, and CT during hepatic arteriography (CTHA) as intra-arterial contrast-enhanced imaging. Among these techniques, CTHA is the best suited to the understanding of the complex hemodynamic interactions in an organ with a dual blood inflow since it depicts both the lesions and the livers free from portovenous enhancement and also avoids improper scan delays that occasionally occur in dynamic contrast-enhanced CT or MR imaging.

### 2.3. Radiological Evaluations of Tumor Blood Inflows and Grades of the Tumors

**Figure 4:**
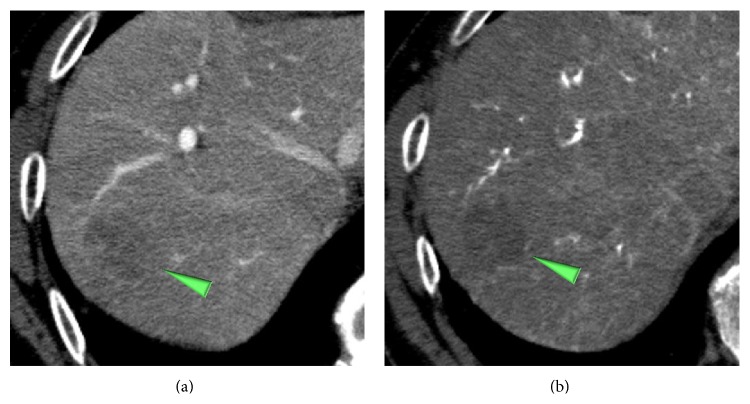
A high grade dysplastic nodule in a 65-year-old man. (a) CTAP image shows a hypoattenuating nodule (arrowhead). (b) The nodule shows hypoattenuating on CTHA image (arrowhead).

**Figure 5:**
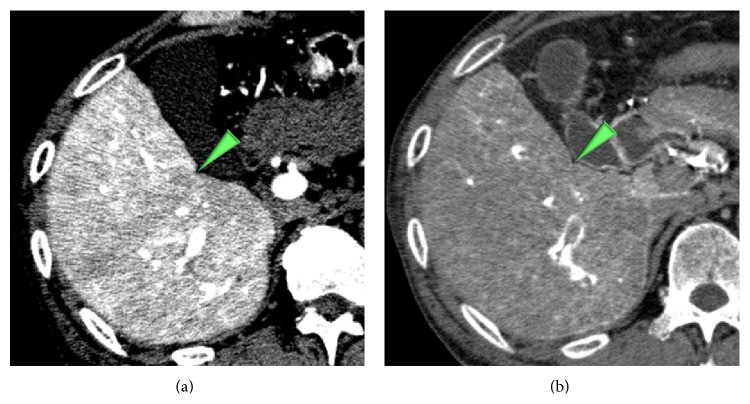
An early HCC in a 76-year-old man. (a) CTAP image shows a hypoattenuating nodule (arrowhead). (b) The nodule shows isoattenuating (= invisible) on CTHA image (arrowhead).

**Figure 6:**
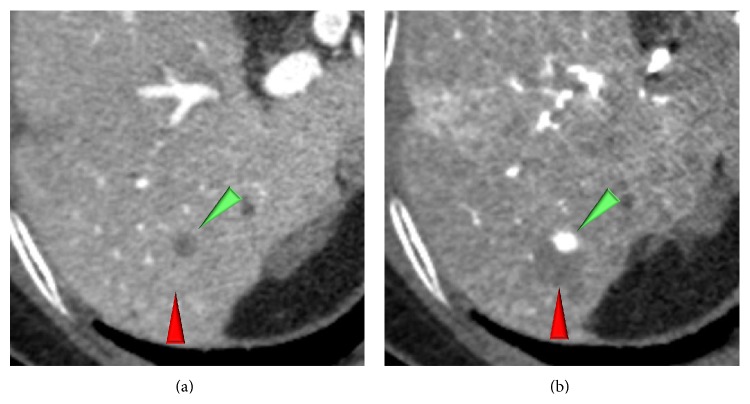
A high grade dysplastic nodule (red arrowheads) harboring a progressed HCC (green arrowheads) in nodule-in-nodule fashion in a 78-year-old woman. (a) CTAP shows a tiny hypoattenuating nodule in an isoattenuating (= invisible) nodule. (b) The tiny nodule shows hyperattenuating within a hypoattenuating nodule on CTHA image.

Imaging techniques illustrate the relations, mentioned in “Section 2.1”, between tumor blood inflows and histological grades in carcinogenic hepatocyte nodules [14, 20, 21]. The attenuations of the nodules on CTAP decrease during dedifferentiation of the nodules: low grade dysplastic nodules show isoattenuating; high grade dysplastic nodules and early HCCs show isoattenuating or slightly hypoattenuating; progressed HCCs show hypoattenuating(Figures 4 and 5). The attenuations of the nodules on CTHA first decrease and then increase: low grade dysplastic nodules show isoattenuating or slightly hypoattenuating; high grade dysplastic nodules and early HCCs show hypoattenuating or isoattenuating; progressed HCCs exclusively show hyperattenuating (Figures 4 and 5). It is noteworthy that high grade dysplastic nodules and/or early HCCs harboring progressed HCCs in nodule-in-nodule fashion are demonstrated on CTAP as isoattenuating or slightly hypoattenuating nodules carrying internal definite hypoattenuating foci, but as hypo- or isoattenuating nodules containing hyperattenuating nodules on CTHA (Figure 6).

Tumor arterial inflows via unpaired arteries cannot be differentiated from those via hepatic arteries by radiological imaging. However, when an isointense nodule on CTHA is depicted as a hypointense nodule on CTAP, the nodule can be interpreted as receiving arterial inflows via unpaired arteries and on the final stage of dysplastic nodule just before progressed HCC (Figure 3).

Although CTAP and CTHA are rather invasive, they are applied as principal techniques for diagnoses of carcinogenic hepatocyte nodules because intravenous contrast enhanced CT is relatively insensitive not only for detection but also for the characterization of these nodules [22, 23]. It is reported that seven dysplastic nodules were categorized as hypoattenuating or isoattenuating on arterial phase CT, but another dysplastic nodule was hyperattenuating; therefore, incorrectly categorized as progressed HCC; 31 progressed HCCs were hyperattenuating and correctly categorized but one progressed HCC was incorrectly categorized as dysplastic nodule [22]. These results correspond with another report [23].

Evaluation of arterial inflows of dysplastic nodules by dynamic contrast enhanced MR imaging is often difficult because dysplastic nodules are generally hyperintense on noncontrast T1-weighted images [24–26]. However, a progressed HCC harbored by dysplastic nodule is depicted as a hyperintense nodule within a hypointense or isointense nodule on arterial phase images.

### 2.4. Tumor Blood Inflows and Outcomes of the Tumors

**Figure 7:**
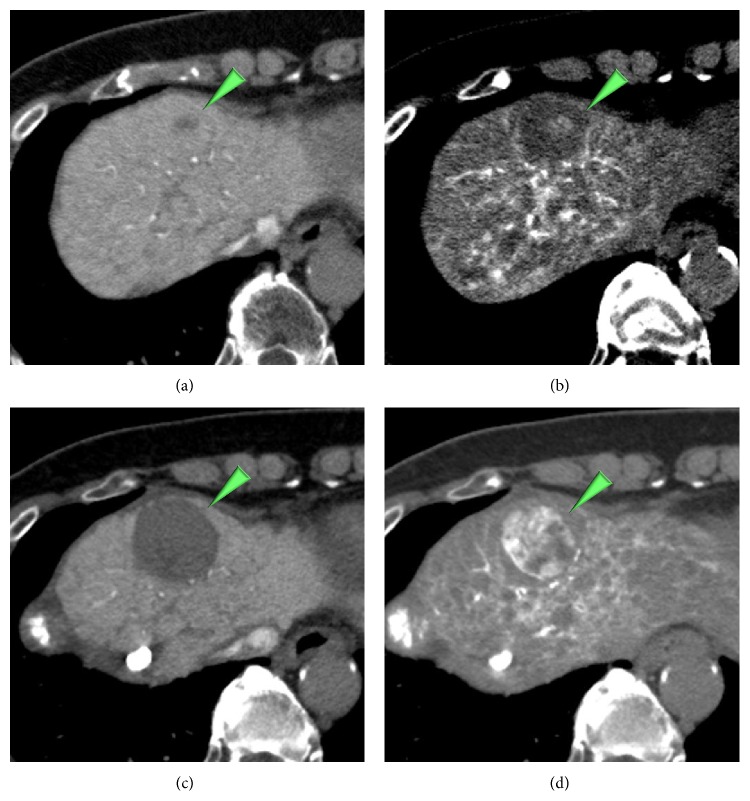
A nodule carrying hypoattenuating focus on CTAP (a) which showed hyperattenuating on CTHA (b) in an 80-year-old woman became entirely hypervascular progressed HCCs ((c), (d)) after 13 months later.

**Figure 8:**
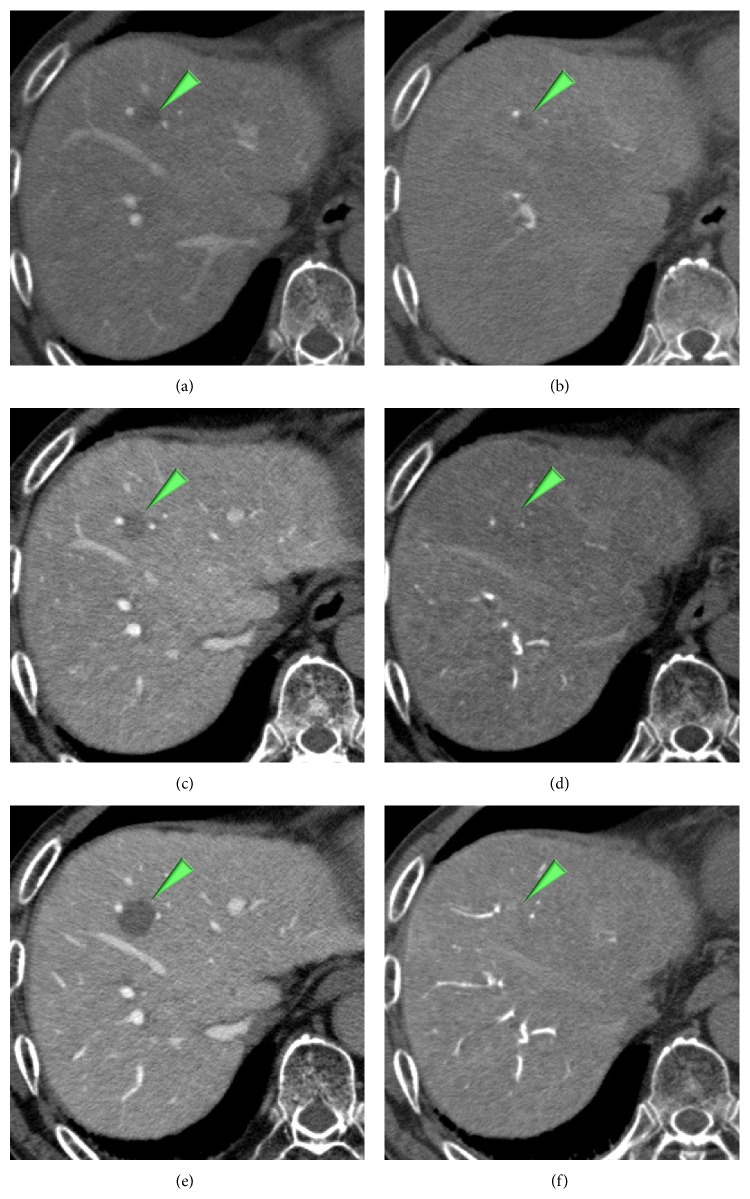
A slightly hypoattenuating nodule on CTAP (a) and CTHA (b) in an 81-year-old woman shows hypoattenuating on CTAP (c) and isoattenuating (d) on CTHA after 16 months later. The nodule shows hypoattenuating on CTAP (e) and slightly hyperattenuating on CTHA (f) after 35 months later from the baseline ((a), (b)).

The outcomes of the carcinogenic hepatocyte nodules can be predicted when both portovenous and arterial inflows are evaluated by CTAP and CTHA, respectively, [15]. No nodules which are isoattenuating on both CTAP and on CTHA became progressed HCCs; approximately 30% of the nodules showing slightly hypoattenuating on both CTAP and CTHA became progressed HCCs after two years. On the other hand, approximately 90% of the nodules carrying hypoattenuating foci on CTAP and hyperattenuating foci on CTHA became entirely hypervascular progressed HCCs after two years (Figure 7). Reduced portal blood flow in the nodule on CTAP is one of the most important predictors for development of progressed HCC (Figure 8) [27].

## 3. Tumor Blood Outflows and Hepatocarcinogenesis

### 3.1. Pathology of Tumor Blood Outflows

**Figure 9:**
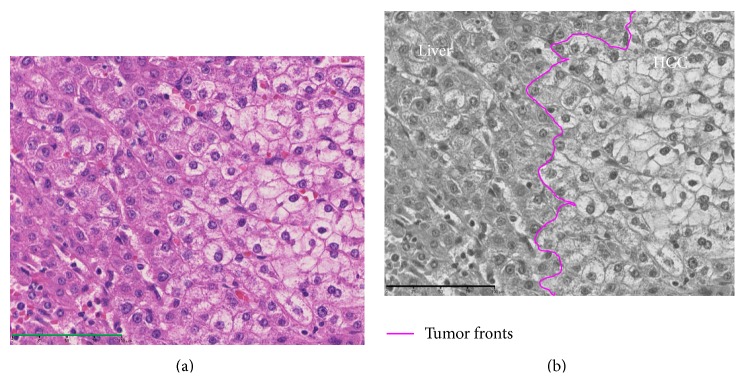
Photomicrograph of tumor front of a high grade dysplastic nodule showing replacing growth stained with hematoxylin and eosin (Bar = 100 *μ*m) (a) and converted monochrome image of the same photomicrograph in conjunction with annotations of colored fronts (b).

**Figure 10:**
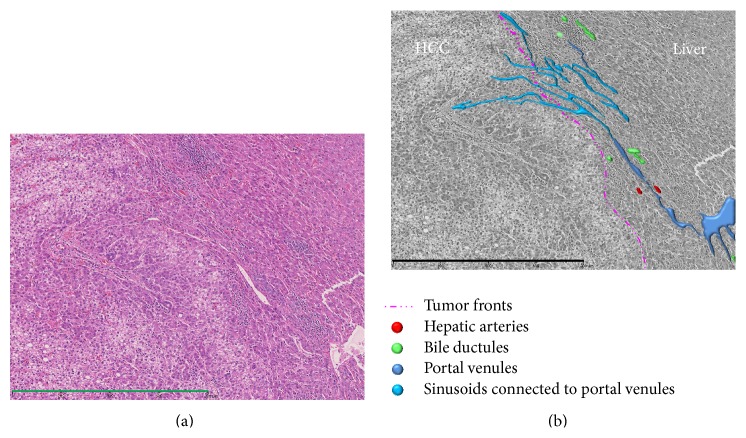
Photomicrograph of tumor front of a progressed HCC showing compressing growth without capsule stained with hematoxylin and eosin (Bar = 800 *μ*m) (a) and converted monochrome image of the same photomicrograph in conjunction with annotations of colored vessels (b). Intranodular capillarized sinusoids connected directly to the surrounding hepatic sinusoids and partly to extranodular portal veins.

**Figure 11:**
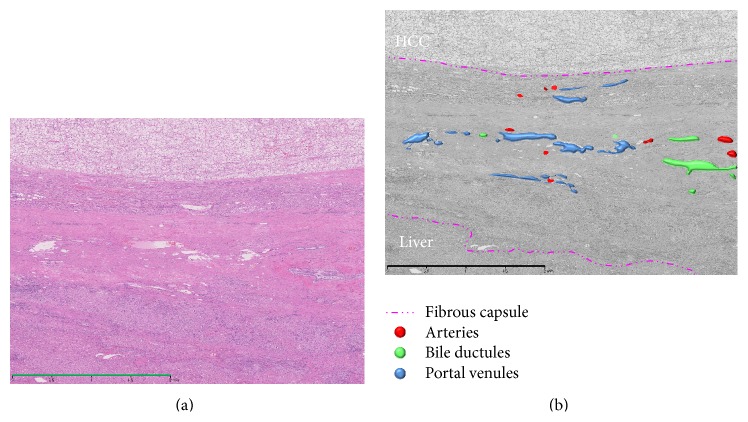
Photomicrograph of tumor front of a progressed HCC showing compressing growth with capsule stained with hematoxylin and eosin (Bar = 2000 *μ*m) (a) and converted monochrome image of the same photomicrograph in conjunction with annotations of colored vessels (b). Intranodular capillarized sinusoids connected to portal venules within the capsule but not directly to the surrounding hepatic sinusoids.

On histology, tumor blood outflows can be speculated through the connections between the vessels within the tumors (capillarized intratumoral sinusoids) and those in the surrounding livers [28]. There are three types of growth patterns in HCCs, namely, replacing growth (Figure 9), compressing growth without capsule (Figure 10), and that with capsule (Figure 11). Replacing growth is seen both in hypovascular early HCCs and in some well differentiated progressed HCCs. Hypovascular early HCC with replacing growth carry more hepatic veins in and around themselves connected with intratumoral blood sinusoids than hypervascular progressed HCCs. Nodules with compressing growth can be classified into two types: nodules without capsule and those with capsule. The former type carries intranodular capillarized sinusoids connected directly to the surrounding hepatic sinusoids and partly to extranodular portal veins. The latter carries intranodular capillarized sinusoids connected to portal venules within the capsule but not directly to the surrounding hepatic sinusoids. Intra- or perinodular hepatic venules decrease in accordance with the grade of malignancy of the nodules and usually no intratumoral hepatic venules are observed in encapsulated HCCs. In poorly differentiated HCCs with invasive growth to the surrounding liver, these connections are not well analyzed and may be more complicated.

### 3.2. Imaging Techniques for Evaluations of Tumor Blood Outflows

There are five imaging techniques for evaluations of tumor blood outflow: single-level dynamic CTHA [29, 30], biphasic CTHA [31], contrast sonography with perfluorobutane microbubbles [32], intravenous dynamic contrast enhanced CT, and intravenous dynamic contrast enhanced MR imaging [26, 33, 34]. Single-level dynamic CTHA, the first technique which demonstrated the tumor blood outflow ever, is the principal technique kinetically depicting both inflow and outflow at one time with the highest temporal resolution. Images can be obtained with 30- to 40-second continuous acquisition without table increment under intrahepatic arterial injection of a small amount of contrast material, for example, 10 mL of 300–370 mgI/mL at a rate of 1.0 mL/sec. Biphasic CTHA is the second technique for evaluations of tumor blood outflow, which covers the whole liver and multiple lesions. Images are obtained with two whole-liver acquisitions at following delays: 10 seconds after start of intrahepatic arterial injection of contrast material of 30 mL of 300–370 mgI/mL at a rate of 1.0 mL/sec for first scan (early phase CTHA) and 60 seconds after start of injection for second scan (late phase CTHA). We can evaluate the arterial inflows with the first scan and the sequential outflows with the second scan. With the remaining three techniques, contrast sonography with perfluorobutane microbubbles, intravenous dynamic contrast enhanced CT, and MR imaging, we can also estimate the outflows, but the reproducibilities and accuracies of the evaluations are much reduced compared with the former two techniques.

### 3.3. Radiological Evaluations of Tumor Blood Outflows and Growth Pattern of the Tumors

Radiological findings of tumor blood outflows of the carcinogenic hepatocyte nodules depend on the channels of blood drainage: hepatic veins, sinusoid, or portal veins [28]. A nodule shows isoattenuation or slight hypoattenuation on CTAP and early phase CTHA and does not show corona enhancement on late phase CTHA when its main drainage channel is hepatic vein. A nodule shows hypoattenuation on CTAP, hyperattenuation on early phase CTHA, and thin corona enhancement (2 mm or less in thickness) on late phase CTHA when its main drainage channel is sinusoid. A nodule shows hypoattenuation on CTAP, hyperattenuation on early phase CTHA, and thick corona enhancement (more than 2 mm in thickness) on late phase CTHA when its main drainage channel is portal vein.

**Figure 12:**
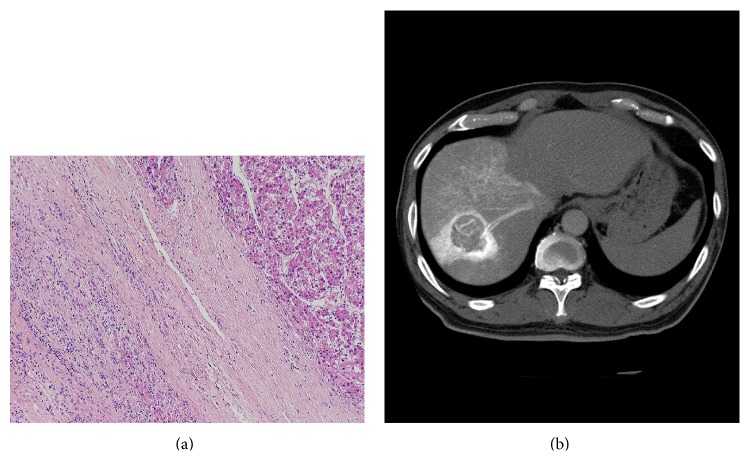
Photomicrograph of tumor front of a progressed well-differentiated HCC showing compressing growth without capsule stained with hematoxylin and eosin (a). The HCC shows thick corona enhancement on late phase CTHA image (b).

**Figure 13:**
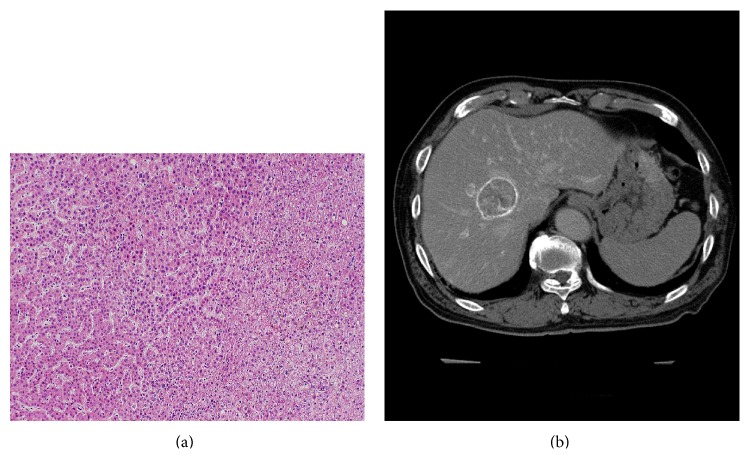
Photomicrograph of tumor front of a progressed well-differentiated HCC showing compressing growth without capsule stained with hematoxylin and eosin (a). The HCC shows thin corona enhancement on late phase CTHA image (b).

**Figure 14:**
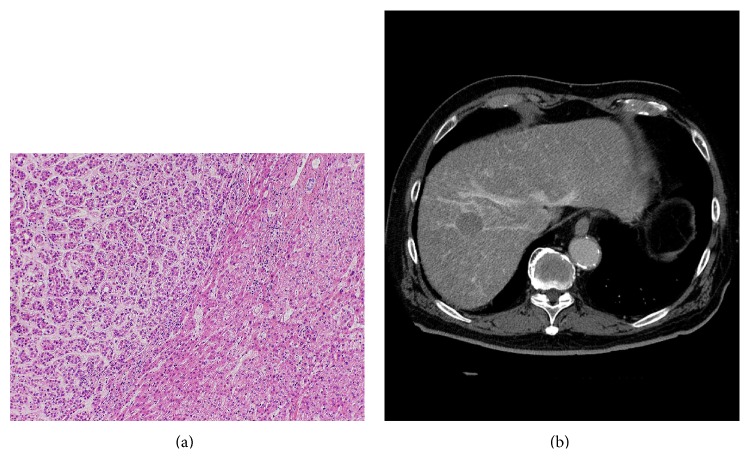
Photomicrograph of tumor front of a progressed well-differentiated HCC showing replacing growth stained with hematoxylin and eosin (a). The HCC does not show any corona enhancement on late phase CTHA image (b).

Findings of corona enhancement relate to the growth patterns of the carcinogenic hepatocyte nodules. Nodules with thick corona enhancement on late phase CTHA commonly show compressing growth with fibrous capsule (Figure 12). Nodules with thin corona enhancement on late phase may show compressing growth without fibrous capsule (Figure 13). Nodules without corona enhancement on late phase CTHA, which appear slightly hypoattenuating on both CTAP and early phase CTHA, show replacing growth (Figure 14).

Growth patterns of the carcinogenic hepatocyte nodules indicate their differentiation. During dedifferentiation, the nodules show replacing growth with indistinct margin at first, then expansile growth without fibrous capsule and finally expansile growth with fibrous capsule [35]. Thus, radiologic evaluations of tumor blood outflows of carcinogenic hepatocyte nodules, especially corona enhancement on late phase CTHA, demonstrate their differentiations as well as their growth pattern.

## 4. Summary

Hemodynamics of carcinogenic hepatocyte nodules depicted with imaging techniques, especially with CTAP and CTHA, are closely related to differentiations, growth patterns, and outcomes of the nodules. Hyperattenuating nodules with thick corona enhancement on CTHA showing hypoattenuation on CTAP are encapsulated progressed HCCs. Those with thin corona enhancement are unencapsulated progressed HCCs with expansile growth. Hypoattenuating or isoattenuating (= invisible) nodules carrying small hyperattenuating areas on CTHA are early HCCs or high grade dysplastic nodules containing tiny progressed HCCs, 90% of which would become wholly progressed HCCs in two years. Isoattenuating nodules on CTHA showing hypoattenuation on CTAP are early HCCs or the final stage of high grade dysplastic nodule just before progressed HCC, 90% of which would become progressed HCCs in two years. Hypoattenuating nodules on CTHA and CTAP are high grade dysplastic nodules, 30% of which would become progressed HCCs in two years. Isoattenuating nodules on CTHA and CTAP detected by other imaging techniques, such as MR imaging, are low grade dysplastic nodule which will seldom change into progressed HCCs. These guides will be helpful in drawing up therapeutic strategies for hepatocyte nodules arising in cirrhosis.
